# Where There's Smoke, There's Fire: Paraneoplastic Autoimmune Limbic Encephalitis

**DOI:** 10.7759/cureus.86628

**Published:** 2025-06-23

**Authors:** Thaw Myint Thu, Sunandan Bhattacharya, Ceris Owen, Dana Chirosca-Vasileiou

**Affiliations:** 1 Cardiology, Medway NHS Foundation Trust, Gillingham, GBR; 2 Acute Medicine, Medway NHS Foundation Trust, Gillingham, GBR; 3 Neurology, Medway NHS Foundation Trust, Gillingham, GBR

**Keywords:** autoimmune, diagnostic puzzle, encephalitis, paraneoplastic, seizure, small cell lung

## Abstract

Autoimmune encephalitis can be stemmed from paraneoplastic, drug-induced, post-infectious, and idiopathic etiologies. It is often but not always associated with neuronal cell surface or intracellular antigens. Regarding encephalitis in the limbic system, the patients mostly present with impairment of cognitive functions with subacute presentation, changes in personalities, memory loss, and seizures. We present a case of a patient who presented with subtle neurological symptoms and later progressed to refractory focal epileptic activities, posing a characteristic sign of limbic encephalitis. He was later diagnosed with small cell lung cancer on further workup. This case poses a classical yet challenging diagnostic puzzle alongside education on how to disentangle patients presenting with de novo neurological signs and symptoms with unremarkable initial screening assessments and investigations in acute settings.

## Introduction

Paraneoplastic neurological syndromes (PNSs) represent immune-mediated neurological manifestations that occur as remote effects of systemic malignancies, independent of direct tumor invasion, metastasis, or treatment-related toxicity. Paraneoplastic neurological syndromes (PNSs) have been reported to affect approximately one in 300 individuals with a diagnosed malignancy. Population-based epidemiological data from a study conducted in Northeastern Italy estimate the incidence of PNSs to range between one and eight cases per 100,000 person-years [1].

Limbic encephalitis (LE) is an inflammatory disease involving the medial temporal lobe, frequently presenting with subacute onset of antegrade amnesia, seizures, or neuropsychiatric symptoms [2]. The etiologies vary, including autoimmune diseases, infections, or paraneoplastic in nature. Paraneoplastic limbic encephalitis (PLE) is most commonly associated with small cell lung cancer (SCLC), testicular germ cell tumor, thymoma, breast cancer, and ovarian teratoma. It has been found that the common autoantibodies associated with LE are anti-Hu (ANNA-1), anti-Ma2, anti-CV2/CRMP5, anti-amphiphysin, anti-Yo (PCA-1), anti-N-methyl-D-aspartate receptor (NMDAR), anti-LGI1, and anti-voltage-gated calcium channels (VGCCs) (P/Q-type and N-type) [3]. The age of onset of paraneoplastic LE depends on the specific underlying cancer. 

There have been ongoing significant challenges with diagnosing LE patients due to rather diverse clinical manifestations, especially in acute and emergency settings, which can also pose a delay in establishing underlying etiologies. Many studies have proven that the prognostication of paraneoplastic LE largely depends on the prompt initiation of treatment for underlying malignancies and immunomodulation therapy for PNSs. Therefore, it is rather critical for acute and emergency clinicians to be having high clinical suspicion of paraneoplastic possibility in dealing with patients presenting with rapid neuropsychiatry decline and attain comprehensive collateral history before concluding the presentation as familiar etiologies such as infection. We present a case of a middle-aged gentleman who presented to an emergency department with a change in personality as the primary complaint and was later diagnosed with paraneoplastic limbic encephalitis due to lung cancer.

## Case presentation

A gentleman in his mid-50s was brought in by ambulance after being found in a self-neglected state, and a change in personality was noticed by his friends and family. He was sent home from his workplace the day prior, and his work colleague visited him to ensure his safety on the day of his admission. The colleague called an ambulance due to concern with one week history of headache reported by the patient and the unusual state of clutter at the place. 

The patient himself did not have any insight on the personality change but reported a global headache for two weeks. He was oriented to people and places but not time. He was working as a professor and was living alone. He did not have any diagnosed medical conditions and was generally healthy.

On clinical examination, the abnormal findings elicited were bilateral increased lower limb tone and brisk reflexes due to suboptimal engagement to undertake the full neurological examination by the patient. He was admitted to the acute medical unit initially with a diagnosis of possible meningoencephalitis and was treated with antibiotics and acyclovir while cerebrospinal fluid (CSF) analysis was processed (Table 1). His capillary blood glucose check at the time of lumbar puncture was 8.9 mmol/L. Due to escalating agitation and ongoing focal seizures, which initially manifested in the acute medical unit, the patient was assessed to pose a significant risk to both himself and healthcare personnel. Consequently, he was sedated, intubated, and transferred to the critical care unit for further management. The initial electroencephalogram (EEG) performed in the critical care setting demonstrated no definitive evidence of epileptic activity.

**Table 1:** Cerebrospinal fluid (CSF) microscopy and analysis.

CSF content parameters (units)	Values/findings	Reference range
White cells (cells/mm^3^)	17	<3
Polymorphs (%)	20%	Not applicable
Lymphocytes (%)	80%	Not applicable
Red blood cells (cells/mm^3^)	6	0–5
Protein (g/L)	0.7	0.15–0.45
Glucose (mmol/L)	4.2	2.8–4.2

The cerebrospinal fluid (CSF) viral polymerase chain reaction (PCR) for Herpes simplex, Varicella zoster, and Enterovirus were negative. The CSF bacterial culture was negative. CSF neuronal antibody tests (anti-contactin-associated protein-like 2 (CASPR2), leucine-rich glioma-inactivated 1 (LGI1), and N-methyl-D-aspartate receptor (NMDAR)) were negative. 

**Figure 1:**
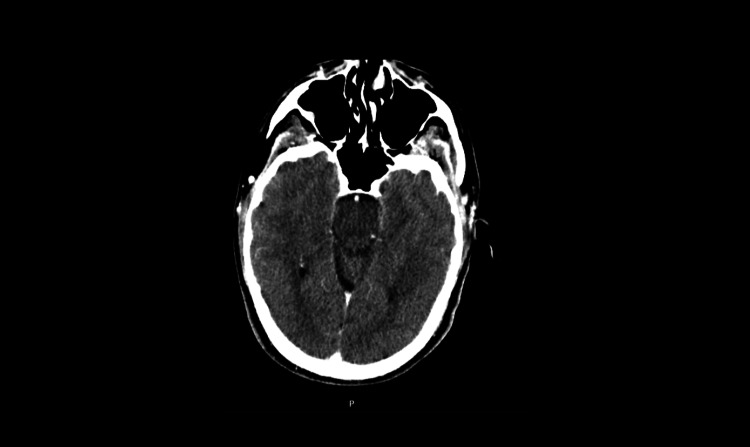
The initial CT head image was reported as unremarkable.

The initial blood tests show neutrophilia with mildly low lymphocyte counts and unremarkable C-reactive protein count. The serum electrolytes, renal function, and liver function tests were normal. The venereal disease research laboratory test (VDRL), HIV, vitamin B12, and folate levels were unremarkable. The serum antinuclear antibodies (ANA), extractable nuclear antigen (ENA), anti-neutrophilic cytoplasmic antibodies (ANCA), anti-Hu, anti-Yo, anti-Ri, collapsin response mediator protein 5 antibodies (CV2/CMRP), zinc finger domain of the intracellular transcription factor (ZiC-4), voltage-gated potassium channels (VGKC), myelin oligodendrocyte glycoprotein antibodies (anti-MOG), anti-glutamic acid decarboxylase antibodies (anti-GAD), dipeptidyl-peptidase-like protein 6 (DPPX) antibody, and neurofilament light chain were negative. Gamma-aminobutyric acid (GABA) type b (GABAb) receptor antibodies were positive. Regarding the imaging studies, the initial CT brain images were reported as unremarkable (Figure 1).

**Figure 2:**
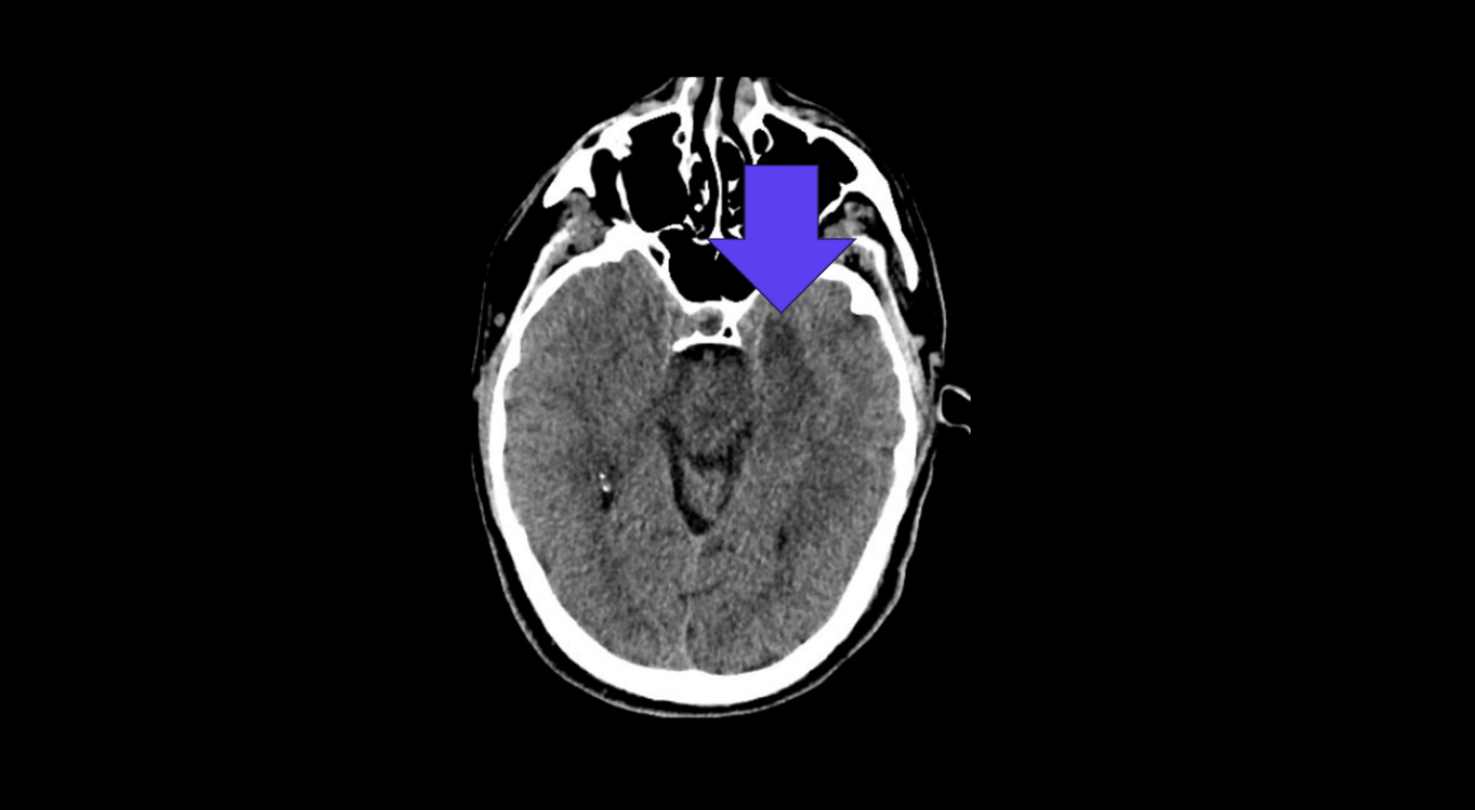
The repeated CT head showing hypodensity of the left medial temporal lobe.

Due to further deterioration with fluctuating consciousness levels, CT head and CT head venogram were conducted five days after the admission day. The hypodensity of the left medial temporal lobe was reported on the repeated CT head (Figure 2).

**Figure 3:**
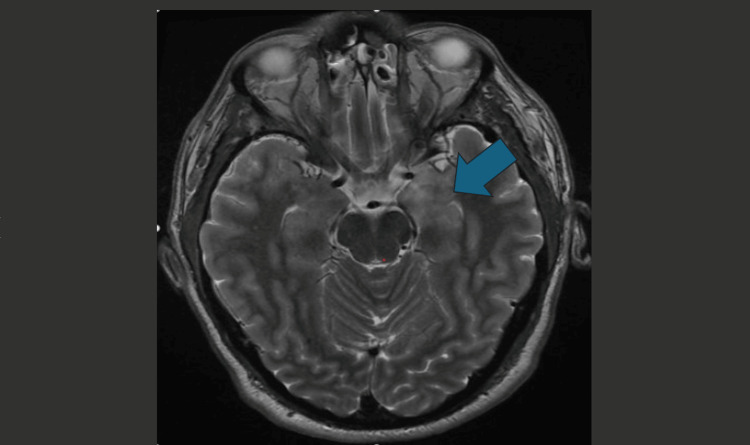
MRI head with contrast showing abnormal signal involving the left medial temporal lobe, eliciting bright T2 and low T1 signal with mild contrast uptake.

MRI head with contrast, MR cranial venogram, and MRI cervical spine with contrast were also arranged to further investigate the prior finding on the CT head. The scans confirmed left medial lobe encephalitis (Figure 3).

He was started on clobazam 10 mg twice daily (BD) and levetiracetam 1.5 gm BD alongside lacosamide 100 mg BD for ongoing focal seizures. The initial diagnosis was changed to autoimmune encephalitis with the investigation results of positive GABAb receptor antibodies and neuroimaging studies. He underwent five sessions of plasma exchange and pulse methylprednisolone IV treatment.

**Figure 4:**
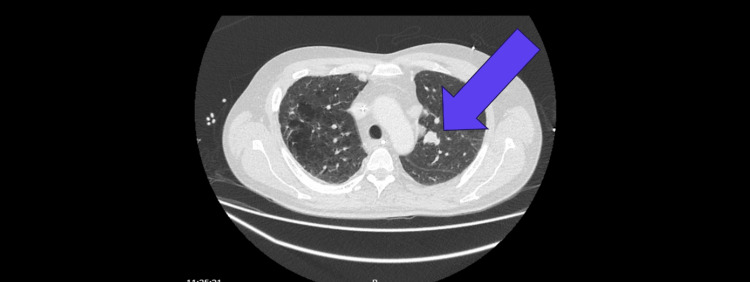
CT (chest, abdomen, and pelvis) showing mediastinal mass (4.2 cm × 1.4 cm × 2 cm) in the pre-vascular area with preserved fat planes.

CT thorax, abdomen, and pelvis with contrast was arranged to rule out paraneoplastic encephalopathy, and he was found to have a mediastinal mass(4.2 cm × 1.4cm × 2cm) in the perivascular area with preserved fat planes (Figure 4). Therefore, he was referred to the pulmonary multidisciplinary meeting. Histology of the lesion shows small cell lung cancer, and the staging was T1B N2/N3 M0. 

**Figure 5:**
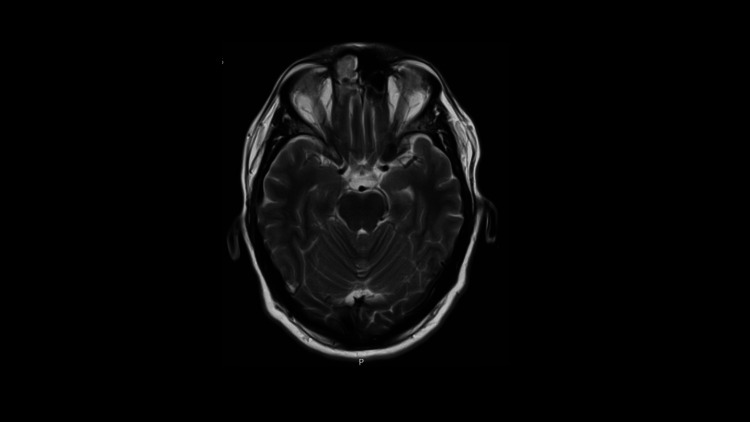
MRI head with contrast showing complete resolution of hyperdense areas with normal enhancement.

He responded very well to plasma exchange and treatment with IV pulse methylprednisolone. He stepped down and was discharged with a weaning dose of oral prednisolone. Antiepileptic agents, levetiracetam 1.5 gm BD and lacosamide 100 mg BD, were continued. His neuropsychiatric presenting symptoms have resolved. The MRI brain performed eight months after the initial presentation shows complete radiological resolution of the previously noted abnormalities (Figure 5).

He was then commenced on chemotherapy (with etoposide and carboplatin) as well as radiotherapy. He continues to have regular surveillance scans for follow-up of lung cancer. His most recent scan shows stable disease with no signs of cancer spread.

## Discussion

This case illustrates the diagnostic convolution of paraneoplastic limbic encephalitis (PLE), an uncommon but rather significant cause of subacute neuropsychiatric decline. The patient, a previously healthy male in his 50s, presented with cognitive impairment, personality changes, and focal seizures that reflect the involvement of limbic structures, such as the hippocampus, amygdala, and cingulate gyrus [4]. The bilaterally increased tone and brisk reflexes in the lower limbs suggested bilateral central nervous system involvement, though initially non-specific to localize a particular group of etiologies. 

The patient’s initial management for suspected meningoencephalitis, including empirical treatment with antibiotics and acyclovir, was appropriate given the acute onset, confusion, and presence of a global headache with no prior diagnosed medical conditions. Infective causes, such as Herpes simplex virus (HSV) encephalitis, are common mimics of autoimmune encephalitis and must be promptly excluded through CSF analysis and neuroimaging studies [5]. 

However, the patient’s rapid deterioration, escalating agitation, and focal seizures despite treatment for infective causes raised concern for a more complex underlying pathology. The eventual diagnosis of PLE underscores the importance of considering autoimmune and paraneoplastic causes in patients with subacute encephalopathy, behavioral changes, and seizures, especially when initial infective and metabolic investigations are unremarkable, as observed in the reported case. 

Diagnosis of PLE is supported by neuroimaging studies (typically MRI brain showing T2/fluid-attenuated inversion recovery (FLAIR) hyperintensities in the medial temporal lobes), CSF findings (mild lymphocytosis, elevated protein), and detection of onconeural antibodies such as anti-Hu, anti-Ma2, or anti-CV2/CRMP5. Notably, antibody-negative PLE may still occur and is diagnosed based on clinical and radiological features, particularly in the context of a newly diagnosed cancer [6]. 

In this case, the neurological presentation preceded the cancer diagnosis, and further investigations revealed the diagnosis of lung malignancy. This aligns with literature reporting that in up to 60% of cases, neurological symptoms are the initial manifestation of an occult tumor [7]. Prompt tumor identification and treatment, along with immunosuppressive therapies (e.g., steroids, IVIG, and plasma exchange), are crucial for improving neurological outcomes. 

This case emphasizes the importance of maintaining a high index of suspicion for PLE in patients with rapidly progressive cognitive and behavioral symptoms. Early multidisciplinary input, including neurology, oncology, and critical care, is vital. Moreover, it highlights the neurological system's role as a sentinel in uncovering systemic malignancies. 

## Conclusions

This case underscores the importance of having a broad and comprehensive approach in formulating the differential diagnoses of patients presenting to acute settings with rather indeterminate manifestations. In dealing with clinically suspected encephalitis cases, undertaking comprehensive clinical assessments, vigilant neuroimaging studies, and meticulous screening of neuronal antibodies in addition to post-diagnostic assessment of associated etiologies would reduce long-term permanent neurological sequelae of the condition as well as negative consequences of the underlying malignant causes. Timely diagnostic evaluation--including serum autoantibody panels, cerebrospinal fluid (CSF) analysis, and neuroimaging studies, particularly brain MRI and imaging studies aimed at identifying underlying neoplastic etiologies--is essential for guiding appropriate therapeutic interventions, such as immunomodulatory therapies and oncological treatment strategies. Last but not least, attaining accurate and thorough collateral history in dealing with any acute or subacute neuropsychiatric decline presentations will help reach the diagnosis with less delay in frontline healthcare settings. 
